# Epileptogenesis in organotypic hippocampal cultures has limited dependence on culture medium composition

**DOI:** 10.1371/journal.pone.0172677

**Published:** 2017-02-22

**Authors:** Jing Liu, Yero Saponjian, Mark M. Mahoney, Kevin J. Staley, Yevgeny Berdichevsky

**Affiliations:** 1 Department of Electrical and Computer Engineering, Lehigh University, Bethlehem, Pennsylvania, United States of America; 2 Department of Neurology, Massachusetts General Hospital, Boston, Massachusetts, United States of America; 3 Harvard Medical School, Boston, Massachusetts, United States of America; 4 Bioengineering Program, Lehigh University, Bethlehem, Pennsylvania, United States of America; University of Modena and Reggio Emilia, ITALY

## Abstract

Rodent organotypic hippocampal cultures spontaneously develop epileptiform activity after approximately 2 weeks in vitro and are increasingly used as a model of chronic post-traumatic epilepsy. However, organotypic cultures are maintained in an artificial environment (culture medium), which contains electrolytes, glucose, amino acids and other components that are not present at the same concentrations in cerebrospinal fluid (CSF). Therefore, it is possible that epileptogenesis in organotypic cultures is driven by these components. We examined the influence of medium composition on epileptogenesis. Epileptogenesis was evaluated by measurements of lactate and lactate dehydrogenase (LDH) levels (biomarkers of ictal activity and cell death, respectively) in spent culture media, immunohistochemistry and automated 3-D cell counts, and extracellular recordings from CA3 regions. Changes in culture medium components moderately influenced lactate and LDH levels as well as electrographic seizure burden and cell death. However, epileptogenesis occurred in any culture medium that was capable of supporting neural survival. We conclude that medium composition is unlikely to be the cause of epileptogenesis in the organotypic hippocampal culture model of chronic post-traumatic epilepsy.

## Introduction

Organotypic hippocampal cultures are increasingly used as an in vitro model of chronic epilepsy [1–3]. This model recapitulates critical features of the development of epilepsy, or epileptogenesis. These features include the latent period between trauma (brain slicing) and the appearance of spontaneous electrical seizures, axon sprouting, and seizure-dependent cell death [4, 5]. Epilepsy progression in this model can be monitored with chronic imaging, electrical recordings, or by assays of biochemical biomarkers of seizure activity and cell death [4–6]. Organotypic cultures thus provide an easy-to-access in vitro platform that can be used for studies of epileptogenesis or for antiepileptic drug discovery [5–9].

Brain insults, including traumatic brain injury, trigger a series of changes at molecular, cellular, and network levels, that can cause epilepsy [10, 11]. It has been hypothesized that the triggering event of epileptogenesis in organotypic cultures is the trauma of brain slice preparation [12]. In cultures, this trauma results in axonal and dendritic reorganization [5–7, 13, 14] and glial activation [15, 16] that are associated with epileptogenesis. In this view, organotypic cultures are an in vitro analogue of animal models of traumatic brain injury-induced epileptogenesis [17–19]. However, unlike brain tissue in vivo, organotypic slices are maintained in an artificially controlled environment. It may be possible that epileptogenesis in organotypic cultures is driven by artificial environmental factors. Here, we test this hypothesis by focusing on the most artificial aspect of organotypic culture environment: the culture medium.

Mammalian tissues must be bathed in a mixture of metabolic substrates, hormones and growth factors (culture medium) to maintain them in vitro longer than 24 hours. Early versions of culture media were often supplemented by actual blood-derived serum [20, 21]. Since concentrations of hormones and metabolites can sometimes vary significantly in samples of animal serum, fully chemically-defined media were developed to enhance reproducibility [22, 23]. We have previously used both serum-supplemented and chemically defined media to maintain organotypic hippocampal cultures, and found that epileptogenesis occurs in both types of media [3, 4]. One concern is that chemically defined culture medium is based on the composition of blood plasma rather than cerebrospinal fluid (CSF). Epileptogenesis in vivo may be enhanced by the opening of the blood-brain barrier (BBB) after brain injury [24, 25]. This enhancement is thought to arise from direct exposure of brain tissue to components of blood that normally do not cross BBB, or to compounds that are present in blood at different concentration than in CSF [26, 27]. Therefore, it may be possible that epileptogenesis in organotypic cultures is not triggered by the trauma of dissection, but by exposure of hippocampal tissue to a cocktail of compounds that are present at much lower concentration or not present at all in normal CSF.

Organotypic slices are prepared from perinatal (postnatal day 7) rodent brain, so the culture media is typically that used for the culture of postnatal neurons (Neurobasal-A, the NeurA column in Table 1) with B27 supplement (Table 2). It can be readily seen that concentrations of glucose, potassium, and magnesium in Neurobasal-A are substantially different than those found in CSF (Table 1, column 3) [28–30]. Some of these differences, such as increased potassium and decreased calcium and magnesium concentrations, may contribute to in vitro hyperexcitability [26, 31]. In addition, many amino acids are contained in Neurobasal-A at significantly higher concentrations than in CSF [32–35]. Altered concentrations of amino acids such as glycine, serine, leucine, isoleucine, valine, phenylalanine and others are found in metabolic epilepsies [36, 37], and might also play a role in development of spontaneous epileptiform activity in organotypic cultures. Neurobasal-A medium is usually supplemented with B27, which contains Bovine Serum Albumin (BSA), insulin, transferrin, progesterone, putrescine, and selenium along with others for a total of 20 components (Table 2). These components may also play a role in epileptogenesis [38].

**Table 1:** Composition of Neurobasal-A, customized medium (CST) and CSF based medium (CBM).

Components	Concentration (mM)				Components	Concentration (mM)			
	NeurA	CST	CBM			NeurA	CST	CBM	
**Essential Amino Acids**					**Vitamins**				
L-Arginine hydrochloride	0.40	0.40	0.40	a	Biotin	0.016	0.016	0.016	b
L-Asparagine·H_2_O	0.0055	-	-		Choline chloride	0.029	0.029	0.029	b
L-Cysteine	0.26	0.25	0.25	a	D-Calcium pantothenate	0.0084	0.0084	0.0084	b
L-Histidine hydrochloride·H_2_O	0.20	0.20	0.20	a	Folic Acid	0.0091	0.0091	0.0091	b
L-Isoleucine	0.80	0.79	0.79	a	Niacinamide	0.033	0.033	0.033	b
L-Leucine	0.80	0.79	0.79	a	Pyridoxine hydrochloride	0.020	-	-	
L-Lysine hydrochloride	0.80	0.81	0.81	a	Riboflavin	0.0011	0.0011	0.0011	b
L-Methionine	0.20	0.20	0.20	a	Thiamine hydrochloride	0.012	0.012	0.012	b
L-Phenylalanine	0.40	0.40	0.40	a	Vitamin B12	0.000005	-	-	
L-Threonine	0.80	0.81	0.81	a	i-Inositol	0.040	-	-	
L-Tryptophan	0.08	0.08	0.08	a	**Inorganic Salts**				
L-Tyrosine	0.40	0.40	0.40	a	Calcium Chloride (CaCl_2_)	1.80	1.80	2.00	*
L-Valine	0.80	0.80	0.80	a	Magnesium Chloride (MgCl_2_)	0.81	0.81	1.30	*
**Non-essential Amino Acids**					Potassium Chloride (KCl)	5.33	5.33	3.50	*
Glycine	0.40	0.40	0.40		Sodium Bicarbonate (NaHCO_3_)	26.19	26.19	25.00	*
L-Alanine	0.023	-	-		Sodium Chloride (NaCl)	68.97	68.97	88.70	c
L-Proline	0.068	-	-		Sodium Phosphate monobasic (NaH_2_PO_4·_H_2_O)	0.91	0.91	1.20	*
L-Serine	0.40	0.40	0.40		Zinc sulfate (ZnSO_4_·7H_2_O)	0.00067	-	-	
**Other Components**					Ferric Nitrate (Fe(NO_3_)_3_·9H_2_O)	0.00025	-	-	
D-Glucose (Dextrose)	25.00	25.00	11.00	*					
HEPES	10.92	10.92	10.92						
Phenol Red	0.07	0.07	0.07						
Sodium Pyruvate	0.23	-	-						

^a^ = from Basal Medium Eagle (BME) amino acids solution B6766 (Sigma)

^b^ = from BME vitamins solution B6891 (Sigma)

^c^ = concentration was adjusted to match osmolarity

- = excluded from medium

* = concentration was according to/approximated CSF composition

**Table 2:** Components of B27 medium supplement.

Components	Concentration (mg/L)^†^	Components	Concentration (mg/L)^†^
**Vitamins**		**Other components**	
Biotin		D-Galactose	
DL Alpha Tocopherol Acetate		Ethanolamine HCl	
DL Alpha-Tocopherol		Glutathione (reduced)	
Vitamin A (acetate)		L-Carnitine HCl	
**Proteins**		Linoleic Acid	
BSA, fatty acid free Fraction V	250	Linolenic Acid	
Catalase		Putrescine 2HCl	16.1
Human Recombinant Insulin	3.5	Sodium Selenite	0.014
Human Transferrin	5	T3 (triodo-I-thyronine)	
Superoxide Dismutase		Progesterone	0.0063
		Corticosterone	

^†^Concentrations of B27 components are not published. The concentrations listed here were used in our media component replacement experiments. Numbers represent final concentrations in culture medium.

In this work, we tested the hypothesis that the composition of culture medium influences epileptogenesis in organotypic hippocampal cultures. To do this, we altered the concentration of individual components of the media and measured the effects on epileptogenesis in this model.

## Methods

### Culture media preparation

Customized culture media were prepared with different compositions and concentrations of electrolytes, amino acids, and glucose (all from Sigma) as described in the text. The osmolarity of all custom media was matched to Neurobasal-A (240–260 mOsm/kg) by adjusting the NaCl concentration. Unless otherwise indicated, all culture media were supplemented with bovine serum albumin (BSA) (250 mg/L: physiological range of albumin in healthy CSF is 70–266 mg/L [39–42]), insulin (3.5 mg/L), selenium (14 μg/L), from Sigma, and glutaMAX (0.5 mM) and gentamicin (30 mg/L), from Life Technologies. In experiments addressing role of B27 components, concentrations of BSA, insulin and selenium were varied as indicated.

### Organotypic hippocampal slice preparation

Postnatal day 7–8 Sprague-Dawley rat pups (Charles River Laboratories) were housed with the dam and anesthetized via isoflurane inhalation and then euthanized by decapitation on the day of dissection. Hippocampi were dissected and cut into 350 μm slices on a McIlwain tissue chopper (Mickle Laboratory Eng. Co., Surrey, United Kingdom) and placed onto poly-D-lysine (Sigma-Aldrich) coated glass cover slips in 6-well tissue culture plates. Slice cultures were maintained in various culture media at 37°C in 5% CO_2_ on a rocking platform. Medium was changed twice a week. All animal use protocols were approved by the Institution Animal Care and Use Committee (IACUC) at Lehigh University or Massachusetts General Hospital Subcommittee on Research Animal Care and were conducted in accordance with the United States Public Health Service Policy on Humane Care and Use of Laboratory Animals.

### Lactate dehydrogenase (LDH) and Lactate assays

Dead or plasma membrane-damaged cells release LDH enzyme [43–45]. We previously found that the amount of LDH in used culture medium correlates with the number of dead neurons in organotypic hippocampal cultures [4, 5]. Lactate production by organotypic hippocampal cultures is increased during ictal activity (seizures) [5]. LDH and lactate levels in the culture medium and the morphology of cultured slices were analyzed together to determine effects of different medium components, as described in Results and Discussion. LDH and lactate were measured in the spent culture media that was collected twice a week during media changes. Spent media was frozen at -80 degrees Celsius, and then batched measurements were carried out using LDH-Cytotoxicity Detection kits (Roche) and L-lactate Assay kits (Eton Bioscience) respectively, according to the manufacturers’ protocols. Lactate concentrations were calculated relative to known lactate standards, while LDH concentrations were calculated in terms of arbitrary units (a.u.), normalized to the 0–3 DIV average of LDH concentration in control culture supernatant.

### Morphology analysis

Brightfield images were taken on an upright microscope (Olympus) with 4x objective. Cultures’ “health” was evaluated based on three morphological criteria: 1. Blurriness of the culture edge. Blurry edges indicate that the slice has attached well to the polylysine substrate, while distinct edge indicates that the slice has not integrated well with the substrate. Very unhealthy slices become completely detached and float in the culture media. 2. Brightness of the slices. Unhealthy slices appear darker than healthy slices due to accumulation of cellular debris. Brightness was quantified by calculating mean greyscale value of the slice area minus the background in bright field images (Fiji/ImageJ). 3. Integrity and distinctness of neural layers. Healthy slices have well-preserved cytoarchitecture with distinct CA1, CA3 neural layers and dentate gyrus (DG).

### Electrophysiological recordings and data analysis

Cover slips with cultures were transferred to a 35 mm petri dish and placed in an interface chamber perfused with culture medium at 37°C. Extracellular field potential recordings were performed using tungsten microelectrodes (0.1 MOhm) connected to an amplifier (RZ2, Tucker Davis Technologies) fitted with high-impedance multiple-channel pre-amplifier stage (PZ2-64, Tucker Davis Technologies) (band-pass 1 Hz-3 kHz, gain ×1000). Sampling rate was 6 kHz per channel. To record the population activity and multiple unit activity, microelectrodes were placed in CA3 pyramidal cell layer. OpenEx (Tucker Davis Technologies) and Matlab (MathWorks) were used for signal processing and data analysis. Ictal events (electrographic seizures) were defined as paroxysmal events of much larger amplitude than background multiple unit activity and lasting longer than 10 s, including discrete shorter paroxysmal events that occurred with event frequency of at least 2 Hz for at least 10 s.

### Nissl and NeuN staining and image analysis

For Nissl staining, cultures were fixed in 4% paraformaldehyde, permeabilized for 1 h in 0.3% Triton-X on a shaking platform, and stained with propidium iodide (Invitrogen; 1 mg/ml stock) diluted 1:250 in phosphate-buffered saline (PBS) for 5 h. Neurons were identified using NeuN antibodies that specifically recognize the DNA-binding, neuron-specific protein NeuN. Cultures were washed in PBS, and fixed for 2 h in 4% paraformaldehyde. Cultures were then transferred to a 48-well plate, and permeabilized in 0.3% Triton X-100 (Sigma-Aldrich) in PBS for 2 h, then blocked with 10% goat serum in PBS for 1 h, followed by incubation for 24 h in 1:100 anti-NeuN antibody conjugated to Alexa Fluor 555 (Millipore) at +4°C on a shaker. Slices were then washed and mounted for confocal microscopy. Z-stack images were collected on a confocal microscope (Zeiss LSM 510 META, Germany) with 40× objective. Z-stack layers were separated by 2 μm, and slices were imaged over their total depth. Images were then processed in Fiji (ImageJ). Neurons in CA1 and CA3 pyramidal layers were quantified with a counting algorithm modified from existing “3D watershed technique” for counting cell nuclei (ImageJ macro developed by [46]).

### Statistical methods

We used Student’s *t* test for two-variable comparisons, one-way ANOVA with Holm-Sidak *post hoc* analysis for multiple variable comparisons, and z-test for seizure-like event incidence comparison. Number of samples n refers to the number of cultures or the number of cells as indicated.

## Results

**Fig 1:**
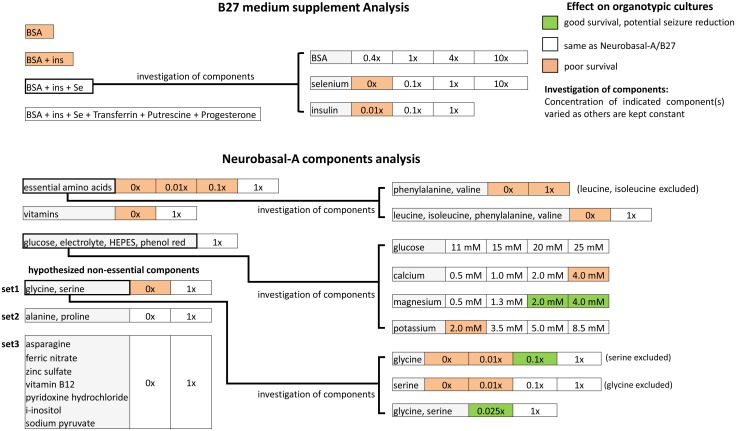
Summary of experiments.

The summary of our experiments is provided in chart form in Fig 1. We focused on identifying components of B27 and Neurobasal-A that were vital for viability of organotypic hippocampal cultures. We then simplified the culture medium, and varied concentrations of essential components to determine effects on epileptogenesis.

### Replacement of B27 supplement with BSA, insulin and selenium

**Fig 2:**
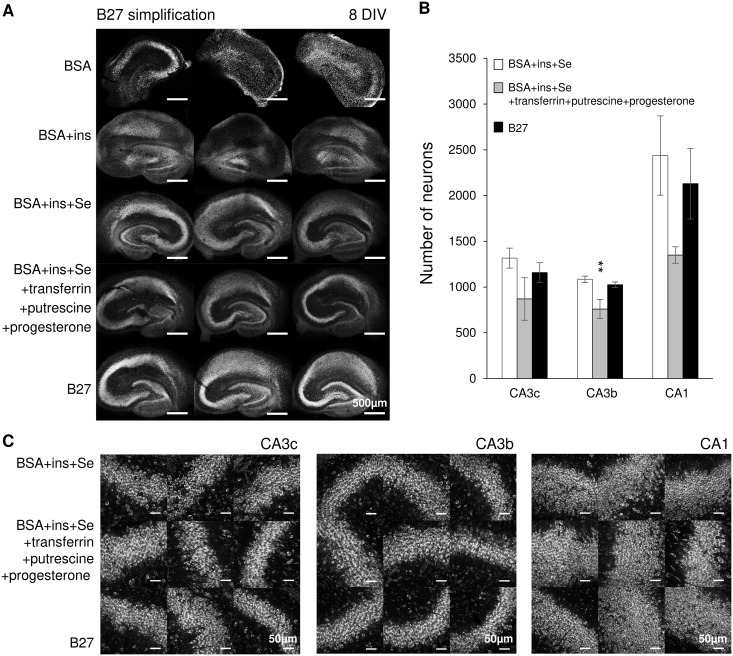
Essential components of B27 supplement. (A) Low magnification confocal images of Nissl staining in cultures at 8 DIV, conditions are indicated on the left side of images, scale bars, 500 μm. (B) Corresponding neuron counts in CA3c, CA3b and CA1. Statistical differences are indicated for comparisons between B27 group versus other groups. Error bars indicate SD. Statistical significance is indicated as **, representing *p* < 0.01. (C) Confocal images of Nissl staining in CA3c, CA3b and CA1, scale bars, 50 μm. n = 3 cultures, each condition.

We hypothesized that only BSA and a subset of components of B27 with concentrations indicated in Table 2 (these components make up the widely used N2 supplement) are essential to maintain viable slice cultures. The following compositions were tested: BSA only; BSA and insulin (BSA + ins); BSA, insulin and selenium (BSA + ins + Se); BSA + insulin + Se + transferrin + putrescine + progesterone; and B27. The concentration of each component is listed in Table 2. Supplements were added to Neurobasal-A medium, which was further supplemented with GlutaMAX and gentamycin as described in Methods. Confocal images taken on 8 DIV showed that cultures kept in medium supplemented with BSA only or with BSA + insulin had missing or fragmented pyramidal layers, indicating poor neuronal survival (Fig 2A). Slices maintained in media with 3 other supplements showed intact pyramidal layers and maintained hippocampal morphology. To further compare these 3 supplements, we quantified the neuron numbers in CA3c, CA3b and CA1. Compared with B27 group, BSA+ins+Se+transferrin+putrescine+progesterone group had a significantly lower number of neurons in CA3b (ANOVA with *post hoc* Holm-Sidak analysis in this and subsequent statistical tests, p = 0.007, n = 3 cultures, each condition) (Fig 2B). BSA + insulin + selenium group showed similar neuron numbers to B27 group in CA3c, CA3b and CA1 (p = 0.222, 0.410, 0.398, respectively, n = 3 cultures, each condition). We conclude that BSA, insulin and selenium were essential and sufficient supplements to Neurobasal-A medium for neuronal survival in organotypic hippocampal cultures until 8 DIV.

### Reduction of selenium and insulin affects cell survival

**Fig 3:**
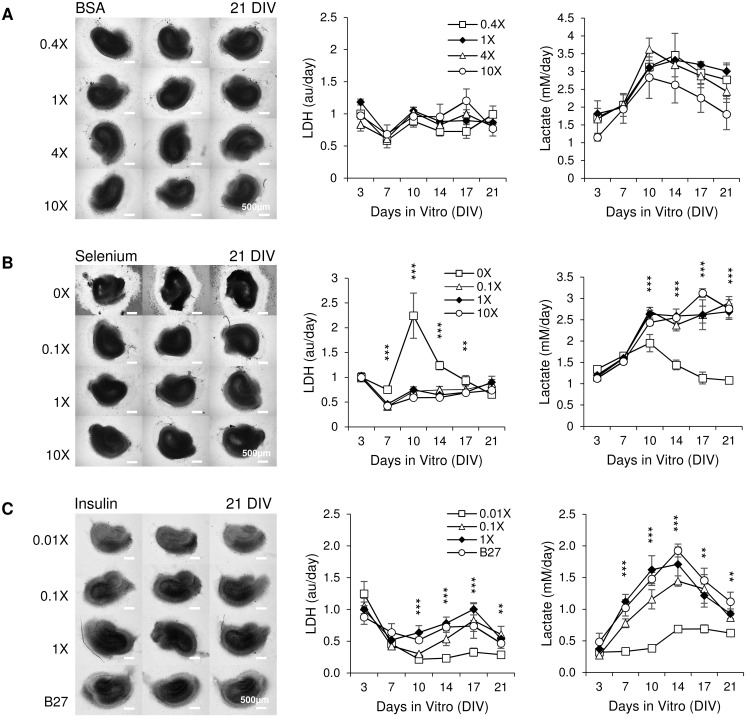
Effects of different concentrations of BSA, selenium, and insulin. (A-C) Left: brightfield images of cultures maintained in media with indicated concentrations of BSA, selenium or insulin, scale bars represent a distance of 500 μm; Right: time course of LDH and lactate concentration in culture supernatant of cultures; n = 3 cultures, each condition. Error bars indicate SD. Significant statistical differences are indicated for comparison of 0X selenium versus 1X selenium and 0.01X insulin versus 1X insulin, with ***p < 0.001, **p < 0.01.

We modified concentrations of each component of the simplified supplement (BSA + insulin + selenium) to investigate their effect on cultures. Following concentrations were tested: for BSA, 0.4x (BSA concentration equal to 0.4 times that listed in Table 2), 1x (BSA concentration equal to that listed in Table 2), 4x (4 times) and 10x (10 times); for selenium, 0x (no selenium), 0.1x (selenium concentration equal to 1/10 of that listed in Table 2), 1x (selenium concentration equal to that listed in Table 2), 10x (10 times); for insulin, 0.01x (insulin concentration is 1/100 of listed), 0.1x (insulin concentration equal to 1/10 of that listed in Table 2), 1x (insulin concentration equal to that listed in Table 2), and B27. The concentration of only one component was varied in each experiment while the other components were included at concentrations found in Table 2. It was not possible to reduce albumin below 100 mg / L due to reductions in the uniformity of wetting of the hippocampal slice surface by the media. Brightfield microscope images were taken at 21 DIV. We measured LDH and lactate concentrations in spent culture media. Data were from medium collected on 3, 7, 10, 14, 17 and 21 DIV. There were no significant differences in gross culture morphology or LDH and lactate concentrations in medium containing different concentrations of BSA (Fig 3A). Cultures in medium with 0x selenium had undistinguishable neural layers, indicating poor survival (Fig 3B). Compared with 1x selenium group, 0x selenium group had significantly higher LDH at 7 DIV, 10 DIV, 14 DIV and 17 DIV (p < 0.001 for 7, 10, 14 DIV, p = 0.01 for 17 DIV, n = 3 cultures, each condition) and significantly lower lactate at 10 DIV, 14 DIV, 17 DIV, 21 DIV (p < 0.001 for all these days, n = 3 cultures, each condition), confirming morphology results and indicating that selenium is essential for culture survival. No differences in morphology or lactate and LDH concentrations were observed between cultures supplemented with 0.1x, 1x, or 10x selenium. Cultures in medium with 0.01x insulin were brighter and smaller than cultures with 0.1x or 1x insulin or B27 (Fig 3C; inverted greyscale values of cultures were 123.41 ± 8.35, 142.13 ± 8.31, 149.47 ± 3.40 and 132.72 ± 7.63 for 0.01x, 0.1x, 1x and B27 group respectively, n = 3 cultures, each condition. A significant difference was found in comparison of 0.01x vs. 1x group, p = 0.007, n = 3 cultures, each condition). Compared with 1x insulin group, 0.01x insulin group had significantly lower LDH from 10 DIV to 21 DIV (p < 0.001 for 10, 14, 17 DIV, p = 0.005 for 21 DIV, n = 3 cultures, each condition) and significantly lower lactate from 7 DIV to 21 DIV (p < 0.001 for 7, 10, 14 DIV, p = 0.007 for 17 DIV, p = 0.003 for 21 DIV, n = 3 cultures, each condition). However, LDH concentration for 0.01x group was moderately higher on DIV 3 than for cultures supplemented with higher concentrations of insulin or with B27. This, together with smaller size of the 0.01x insulin cultures, suggested that more cells died by DIV 3 due to lack of sufficient concentration of insulin, leading to lower lactate production in these cultures later on.

**Fig 4:**
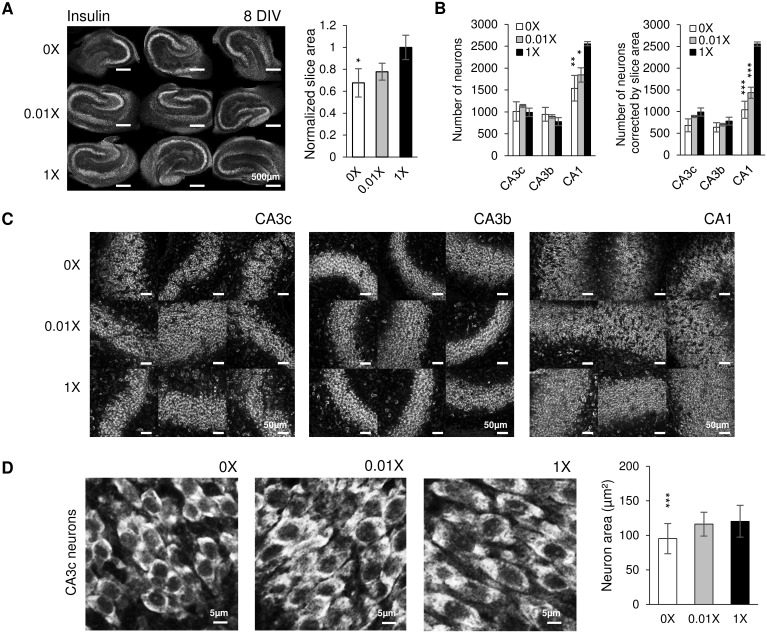
Insulin enhances neuronal survival. (A) Low magnification confocal images of Nissl staining in cultures at 8 DIV, conditions are indicated on the left side of images, scale bars, 500 μm. Chart shows effect of insulin on slice area; (B) Left: neuron counts in CA3c, CA3b and CA1; Right: neuron counts corrected by the normalized slice area. n = 3 cultures, each condition. Statistical differences are indicated for comparison between 1X group and other groups. (C) Representative confocal images of Nissl staining in CA3c, CA3b and CA1, scale bars, 50 μm. (D) Left: representative confocal images of neurons in CA3c, scale bars represent a distance of 5 μm; Right: corresponding cell size in CA3c. n = 30 cells, each condition. Significant statistical differences are indicated for comparison of 0X group versus 1X group. Error bars indicate SD. Significant differences are labeled as *p < 0.05, **p < 0.01 and ***p < 0.001.

We further investigated the influence of insulin concentration on culture survival. We used the following conditions: 0x (no insulin), 0.01x (insulin concentration is 1/100 of that listed in Table 2), and 1x. Confocal images taken on 8 DIV showed that 0x group had smaller slice area compared with other groups (Fig 4A). We measured the slice area and a significant difference was seen between the 0x group and 1x group (p = 0.011, n = 3 cultures, each condition). We quantified the number of neurons in CA3c, CA3b and CA1 (Fig 4B, left chart). However, we found that due to the more compact size of 0x insulin cultures, a larger proportion of the pyramidal cell layer would fit into a single field of view for those cultures compared to 0.01x and 1x cultures. To arrive at a correct relative number of neurons between the cultures, we corrected neuronal counts with normalized total slice area (Fig 4B, right chart). Compared to the 1x group, the 0x group and 0.01x group showed significantly lower neuron numbers in CA1 (before slice area correction: p = 0.002 for 0x group, p = 0.011 for 0.01x group, n = 3 cultures, each condition; after slice area correction: p < 0.001 for both conditions). We inspected the size of individual neurons and found that 0x group and 0.01x group had smaller neurons compared to the 1x group (Fig 4D). A significant difference was seen between the 0x group and 1x group (p < 0.001, n = 30 cells, each condition). We concluded that insulin affects neuronal size, survival and slice size. However, insulin concentrations that were reduced by a factor of 10 from the standard N2 levels had no significant effects on our measures of the health of the slice cultures. In subsequent experiments, we supplemented all media with BSA, insulin and selenium at concentrations listed in Table 2, since these concentrations were optimal for neural survival by DIV 8.

### Identification of essential components of Neurobasal-A

**Fig 5:**
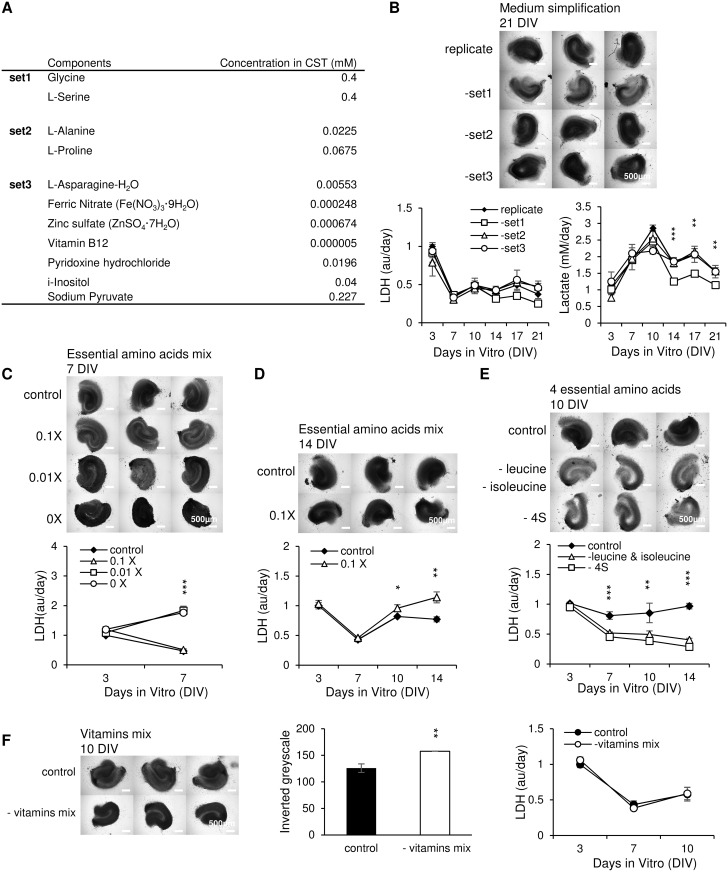
Neurobasal-A simplification. (A) List of hypothesized noncritical components. (B) Left: brightfield microscope images at 21 DIV, scale bars represent a distance of 500 μm; Right: time course of LDH and lactate concentration in spent culture media from cultures treated with replicated NeurA, or NeurA without set1, set2 or set3; n = 3 cultures, each condition. Significance is shown for replicate group versus–set1 group. (C) Cultures maintained in the presence of different concentrations of essential amino acids. Top: photomicrographs at 7 DIV. Scale bars, 500 μm; n = 3 cultures, each condition. Statistical significance is indicated for 0x group and 0.01x group versus control. (D) Cultures maintained in the presence of reduced and control concentrations of amino acids. Images are from 14 DIV; n = 3 cultures, each condition. (E) Evaluation of the removal of 4 metabolic epilepsy-related essential amino acids (4S: leucine, isoleucine, phenylalanine and valine). Images are from 10 DIV; n = 3 cultures, each condition. Statistical differences are shown for experimental groups versus control. (F) Cultures maintained in the presence of 1x (control) and without vitamins (- vitamins mix) and corresponding greyscale and LDH measurements. Images are from 10 DIV; n = 3 cultures, each condition. Error bars indicate SD. Significances are labeled as ***p < 0.001, **p < 0.01 and *p < 0.05.

We grouped the hypothesized non-essential components into three sets listed in Fig 5A. We then replicated Neurobasal-A by combining BME amino acids solution (B6766, Sigma, 1:12.5 dilution) and BME vitamins solution (B6891, Sigma, 1:25 dilution) with other components in Neurobasal-A formulation in appropriate concentrations (Table 1, NeurA columns). Suspected noncritical components were included in the replicate experimental group, or excluded as indicated. Cultures were maintained in replicated NeurA or replicated NeurA with set 1, set 2, or set 3 excluded (-set1, -set2, -set3 experimental groups). Brightfield microscope images taken on 21 DIV showed that cultures without glycine and L-serine (-set1) were significantly brighter than other groups (Fig 5B; inverted greyscale = 152.60 ± 6.74, 110.28 ± 9.29, 148.85 ± 2.98 and 152.65 ± 7.39 for replicate, -set1, -set2 and–set3 group respectively; p < 0.001 for–set1 vs. replicate, n = 3 cultures, each condition). Slices in–set2 and–set3 had morphologies similar to control (replicated NeurA) slices. Group lacking set1 was found to have lower LDH and lactate concentrations than the other three groups from 14 DIV to 21 DIV. Significant differences in lactate between–set1 group and NeurA replicate group appeared on 14, 17, 21 DIV (p < 0.001, p = 0.004, p = 0.007, respectively, n = 3 cultures, each condition). Based on the altered morphology, LDH, and lactate, we conclude that glycine and L-serine play a role in cell survival and activity. On the other hand, exclusion of set2 or set3 had no noticeable effect on culture morphology or LDH and lactate release. In subsequent experiments, we used NeurA medium with the noncritical sets 2 and 3 excluded, which we termed customized medium (CST, Table 1). The role of glycine and L-serine was investigated further in experiments described in the Reduction of non-essential amino acids affects cell survival section.

Based on CST, we generated media with different concentrations of essential amino acids: control (essential amino acid concentrations equal to those listed for CST), 0.1x (essential amino acid concentrations equal to 1/10 of those listed for CST), 0.01x (1/100) and 0x (no essential amino acids). By 7 DIV, cultures in 0x group were significantly darker than control cultures, indicating poor survival (Fig 5C; inverted greyscale values were 135.19 ± 3.42, 124.77 ± 3.87, 144.46 ±15.32 and 170.53 ± 10.56 for control, 0.1x, 0.01x and 0x group respectively; p = 0.001 for 0x vs. control group, n = 3 cultures, each condition). Neural layers became undistinguishable in 0x cultures by 7 DIV. 0.01x and 0x group had significantly higher LDH than control at 7 DIV (p < 0.001, n = 3 cultures, each condition). We conclude that concentrations of essential amino acids lower than 1/100 of what is found in Neurobasal-A are insufficient for culture survival. We then compared 0.1x and control groups (Fig 5D). No observable differences in culture morphology were found in microscope images at 14 DIV. However, 0.1x group had significantly higher LDH than control at 10 DIV (p = 0.03, n = 3 cultures, each condition) and 14 DIV (p = 0.006, n = 3 cultures, each condition). Thus, we conclude that reduction of all essential amino acid concentrations was detrimental to culture survival.

We then examined the possibility that we could reduce seizure activity in organotypic slice cultures by reducing the concentrations of some of the essential amino acids, without increasing cell death. We focused on the amino acids that play a role in metabolic epilepsy: leucine, isoleucine, phenylalanine and valine (Fig 5E). Control cultures were maintained in CST with all essential amino acids, -leucine & isoleucine cultures were maintained in CST without leucine and isoleucine, and -4S cultures were maintained in CST without leucine, isoleucine, phenylalanine, and valine. The cultures of the last two groups had a deformed shape with a hole in the slice center, and much thinner neural layers compared with the control group at 10 DIV. The control group showed significantly higher LDH than the other two groups at 7 DIV (p < 0.001 for both groups), 10 DIV (p = 0.009 for–leucine and isoleucine group, p = 0.035 for -4S group), and 14 DIV (p < 0.001 for both groups). In this case, early cell death in–leucine & isoleucine and -4S cultures (detected by poor morphology of these cultures) resulted in lower LDH at later time-points, due to fewer surviving cells. We conclude that leucine, isoleucine, phenylalanine, and valine are essential for organotypic hippocampal culture viability.

We also excluded all vitamins (-vitamins mix), and compared it with 1x vitamins concentration medium (control). Microscope images at 10 DIV revealed that the -vitamins mix group slices shrank, with significantly darker neural layers than control cultures, despite no significant difference in LDH (Fig 5F; inverted greyscale = 125.77 ± 8.21 and 157.73 ± 0.30 for control and–vitamins mix group respectively, p = 0.005, n = 3 cultures, each condition). We conclude that exclusion of vitamins increased cell death in cultures.

### Reduction of non-essential amino acids affects cell survival

**Fig 6:**
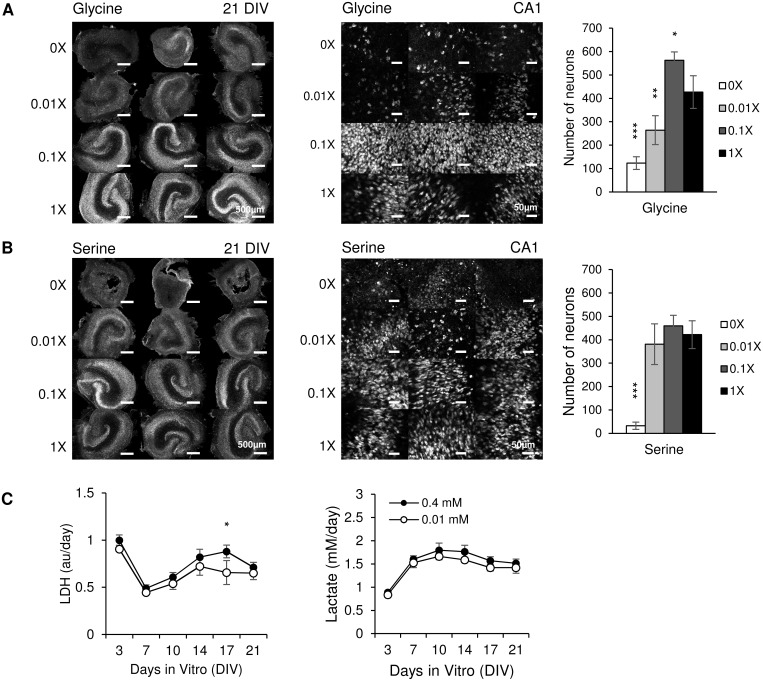
Cultures in media with different glycine and serine concentrations. (A) Left: low magnification confocal images of NeuN staining in cultures at 21 DIV, glycine concentrations are indicated on left side of images, scale bars, 500 μm; Middle: representative confocal images of NeuN staining in CA1, scale bars, 50μm; Right: corresponding CA1 neuron counts; n = 3 cultures, each condition. Statistical differences are indicated for comparisons between 1x group versus other groups. (B) Cultures maintained in different concentrations of serine. Significances are indicated for 0x group versus 1x group; n = 3 cultures, each condition. (C) Lactate and LDH results for cultures treated with combined glycine and serine, n = 3 cultures, each condition. Error bars represent SD. Significant differences are labeled as *p < 0.05, **p < 0.01 and ***p < 0.001.

We investigated glycine and L-serine in more detail. We maintained cultures in media containing different concentrations of glycine or L-serine (only one of those amino acids was present in media described in this paragraph and Fig 6A and 6B. Base concentration (1x) of glycine or L-serine was 0.4 mM as in NeurA. 0x medium contained neither glycine nor L-serine. Confocal images of cultures stained with anti-NeuN neuron-specific antibody were taken at 21 DIV (Fig 6A). Cultures maintained in 0x and 0.01x glycine had very few neurons remaining by 21 DIV. NeuN staining in 0.1x glycine group revealed distinct neuron layers similar to 1x group. Neuronal counts in area CA1 revealed that compared with 1x group, significantly less neurons remained in 0x (p < 0.001, n = 3 cultures, each condition) and 0.01x (p = 0.009, n = 3 cultures, each condition) glycine groups, while more neurons remained in 0.1x (p = 0.041, n = 3 cultures, each condition) group. Cultures maintained in 0x serine group cultures shrank and lost hippocampal morphology, while cultures maintained in 0.01x serine had distinct CA1 and CA3 neuronal layers but few neurons in DG (Fig 6B). In 0.1x and 1x L-serine groups, cultures had well preserved and distinct pyramidal and granule neuron layers (bright NeuN staining). The number of neurons in CA1 was significantly lower in 0x L-serine group (p < 0.001, n = 3 cultures, each condition) compared with 1x group. There were no significant differences between numbers of CA1 neurons in 0.01x, 0.1x and 1x L-serine groups. Based on CA1 neuron counts and preservation of DG, we conclude that concentrations of glycine and L-serine less than 0.1x (0.04mM) were insufficient to support culture survival.

We then examined whether combined glycine and L-serine could support neurons in organotypic cultures at lower concentration than glycine or L-serine alone. We compared lactate and LDH release in media containing 0.01 mM glycine and L-serine and in medium containing 0.4 mM glycine and L-serine (CST medium) (Fig 6C). Slightly less lactate and LDH was released in medium with 0.01 mM glycine and serine compared to medium with 0.4 mM glycine and serine, although results were not statistically significant except for LDH on 17 DIV (p = 0.036, n = 3 cultures, each condition). We conclude that when both L-serine and glycine are included in culture media, concentrations can be reduced to 0.01 mM without adversely affecting cell survival. Slightly higher LDH and lactate release in medium with higher glycine and serine concentrations pointed to a potential pro-epileptic effect of these amino acids.

### Modification of electrolyte and glucose concentrations affects cell survival and ictal activity

**Fig 7:**
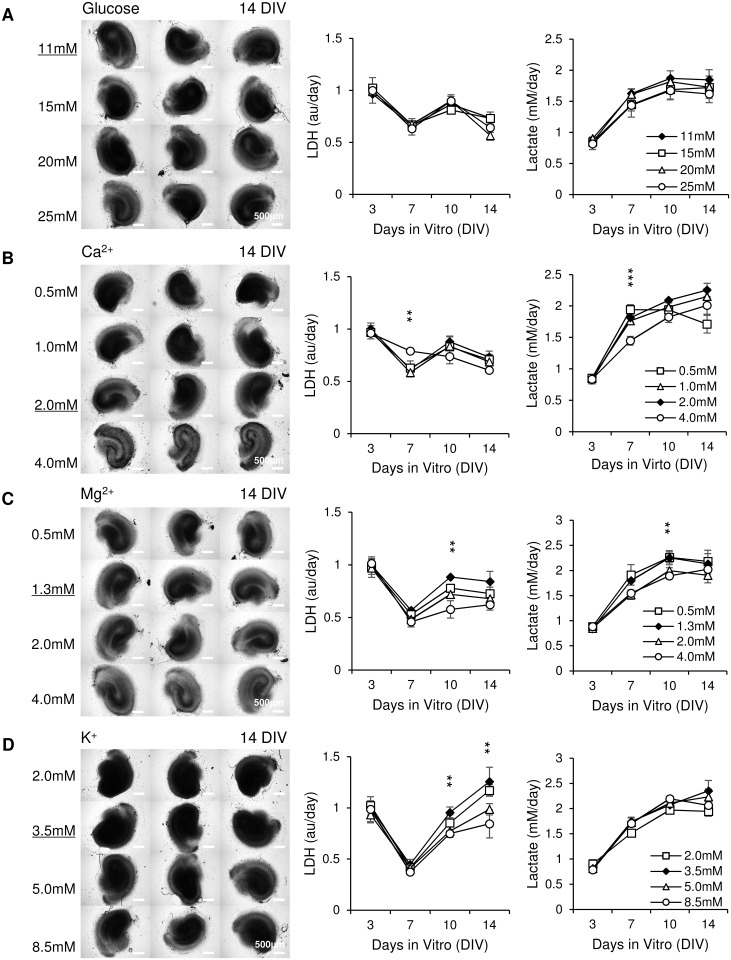
Modification of glucose and electrolyte concentrations affects cell survival and ictal activity. (A-D) Left: brightfield images of cultures maintained in media with indicated concentrations of glucose, Ca^2+^, Mg^2+^, or K^+^ at 14 DIV, scale bars, 500 μm; Right: time course of LDH and lactate release into culture medium. n = 3 cultures, each condition. Error bars indicate SD. Concentrations of ions that correspond to rat CSF are underlined and indicated by solid symbols. Significant statistical differences are indicated for comparison of 4 mM Ca^2+^, 4 mM Mg^2+^ and 8.5 mM K^+^ versus CSF concentrations of corresponding ions., with **p < 0.01 and ***p < 0.001.

Concentrations of glucose, calcium, magnesium and potassium in CST are different from those found in rat CSF. We modified the concentrations of these components to investigate their effect on cultures. The following concentrations were tested: for glucose, 11 mM (glucose concentration used in typical artificial CSF (aCSF) for acute slice experiments [4]), 15 mM, 20 mM, and 25 mM; for calcium, 0.5 mM, 1 mM, 2 mM ([Ca^2+^] used in typical aCSF), 4 mM; for magnesium, 0.5 mM, 1.3 mM ([Mg^2+^] used in typical aCSF), 2 mM, and 4 mM; for potassium, 2 mM, 3.5 mM ([K^+^] used in typical aCSF), 5 mM, and 8.5 mM. Concentration of only one medium component was varied in each experiment; other components were included at concentrations found in CST (Table 1). Brightfield microscope images were taken at 14 DIV. There were no differences in culture morphology or LDH and lactate in media containing different concentrations of glucose (Fig 7A). Cultures in medium with [Ca^2+^] = 4 mM had dark edges around neuron layers, indicating that cell death occurred (Fig 7B). Compared with [Ca^2+^] = 2 mM group, [Ca^2+^] = 4 mM group had significantly higher LDH (p = 0.004, n = 3 cultures, each condition) and significantly lower lactate (p < 0.001, n = 3 cultures, each condition) at 7 DIV, confirming morphology results and indicating that high Ca^2+^ concentration is toxic for organotypic hippocampal cultures. Cultures in medium with [Mg^2+^] = 4 mM had significantly brighter neural layers than cultures in media with lower [Mg^2+^], which is potentially due to lower dead cell accumulation (Fig 7C; inverted greyscale = 144.94 ± 1.19, 146.73 ± 3.43, 141.21 ± 3.60 and 130.20 ± 2.08 for 0.5 mM, 1.3 mM, 2.0 mM and 4.0 mM group respectively; p < 0.001 for 4.0 mM vs. 1.3 mM, and 4.0 mM vs. 0.5 mM, p = 0.04 for 4.0 mM vs. 2.0 mM, n = 3 cultures, each condition). Interestingly, cultures in [Mg^2+^] = 2 mM and [Mg^2+^] = 4 mM groups had significantly lower LDH and lactate release compared with [Mg^2+^] = 1.3 mM group at 10 DIV (p = 0.001, LDH, n = 3 cultures, each condition, and p = 0.006, lactate, n = 3 cultures, each condition). Cultures in media containing [K^+^] = 5 mM and [K^+^] = 8.5 mM group had better morphology (lighter, clearer neuronal layers) than cultures in [K^+^] = 3.5mM, while cultures in [K^+^] = 2 mM group had the worst morphology (dark, neuronal layers not visible) (Fig 7D). [K^+^] = 8.5 mM group released significantly less LDH than [K^+^] = 3.5 mM group at 10 and 14 DIV (p = 0.007 and p = 0.008, respectively, n = 3 cultures, each condition). There were no significant differences found in lactate release by cultures in media with different [K^+^]. Brighter neural layers and lower LDH in [K^+^] = 8.5 mM group indicated that high concentration of K^+^ was neuroprotective (inverted greyscale = 169.73 ± 4.27, 168.07 ± 6.20, 152.08 ± 0.45 and 143.69 ± 4.17 for 2.0 mM, 3.5 mM, 5.0 mM and 8.5 mM group respectively; p < 0.001 for 8.5 mM vs. 2.0 mM, and 8.5 mM vs. 3.5 mM, p = 0.003 for 5.0 mM vs. 2.0 mM, p = 0.006 for 5.0 mM vs. 3.5 mM, n = 3 cultures, each condition). This finding was surprising, since acute application of high [K^+^] was previously found to increase ictal activity in acute hippocampal slice preparations [47]. The potential anticonvulsant effect of elevated [Mg^2+^] in organotypic cultures, evidenced by lower lactate production, is consistent with data from other models [48, 49].

### CSF-based medium can support organotypic hippocampal cultures

**Fig 8:**
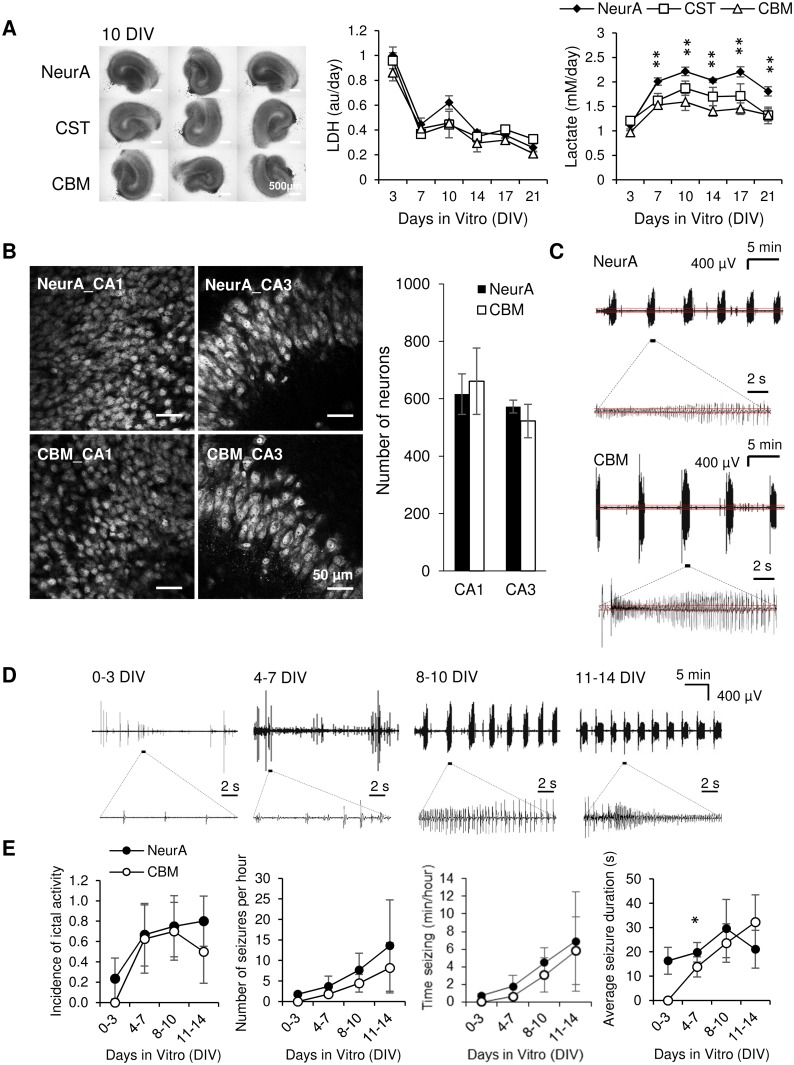
Comparison between NeurA, CST and CBM media. (A) Brightfield images at 10 DIV. Scale bars, 500 μm. Time course of LDH and lactate concentration in culture supernatant; n = 3 each condition per time point. Error bars indicate SD. Statistical significant differences are indicated for CBM and CST versus NeurA, with **p < 0.01. (B) Representative confocal images of CA1 and CA3 area of NeuN-stained organotypic hippocampal cultures at 10 DIV. Scale bars, 50 μm. Neuron counts in CA1 and CA3 are shown on the right; n = 3 each condition. (C) Representative recordings of electrographic seizure-like events in organotypic cultures on 10 DIV. Seizure detail is shown in lower traces. Red lines indicate the threshold for paroxysmal event detection. (D) Representative recordings on different days in vitro in cultures maintained in NeurA, revealing the time course of epileptogenesis in organotypic hippocampal cultures. (E) Electrographic seizures (ictal events) in NeurA and CBM in electrographic recordings of seizing slices. For 0–3 DIV, 4–7 DIV, 8–10 DIV, 11–14 DIV,n = 17, 9, 8, 10 for NeurA, n = 15, 8, 10, 10 for CBM, respectively. n represents the number of cultures. *p < 0.05. Error bars represent 95% confidence intervals for incidence of ictal activity and SD for other graphs.

We hypothesized that changing concentrations of glucose, Ca^2+^, Mg^2+^, and K^+^ simultaneously may produce different effects than changing their concentrations individually. We made another medium, defined as CSF-based medium (CBM). CBM has the same components as CST, but concentrations of electrolytes were changed to reflect concentrations in rat CSF, while glucose concentration was changed to match typical concentration used in aCSF for acute slice experiments [50] (Table 1). Cultures maintained in CST or CBM were compared to cultures maintained in NeurA (Fig 8A). Compared with NeurA slices, CST and CBM cultures showed similar well-preserved hippocampal morphology with clear neuronal layers. No significant differences in LDH release were found between the three experimental groups, confirming morphology results (n = 3 cultures, each condition). On the other hand, significantly lower lactate release in CST and CBM groups were found in comparison to the NeurA group starting from 7 DIV to 21 DIV (at 7 DIV, 10 DIV, 14 DIV, 17 DIV and 21 DIV, for CST versus NeurA, p = 0.003, 0.015, 0.023, 0.012, 0.002, respectively; for CBM versus NeurA, p = 0.002, 0.001, 0.001, 0.002, 0.006, respectively, n = 3 cultures, each condition). Significant differences were also found between CST and CBM groups at 10 DIV and 14 DIV (p = 0.038, and 0.036, respectively, n = 3 cultures, each condition).

Confocal images of NeuN-stained cultures showed that pyramidal neuronal layers were well organized and densely packed in both NeurA and CBM cultures at 10 DIV (Fig 8B). The numbers of surviving neurons were not different between NeurA and CBM cultures in either CA1 (p = 0.663, student *t* test, n = 3 cultures, each condition) or CA3 (p = 0.317, student *t* test, n = 3 cultures, each condition).

To further investigate the reduced lactate production in CBM and CST vs. NeurA culture media, we quantified electrographic seizures (ictal events) in NeurA and CBM cultures from 0 to 14 DIV. Examples of recordings from cultures maintained in NeurA are shown in Fig 8D. To compare effects of NeurA and CBM, data were grouped into four time periods: 0–3 DIV, 4–7 DIV, 8–10 DIV, and 11–14 DIV (at each time point, for NeurA: n = 17 from 6 animals, 9 from 6 animals, 8 from 4 animals, 10 from 4 animals, respectively; for CBM: n = 15 from 6 animals, n = 8 from 6 animals, n = 10 from 3 animals, n = 10 from 4 animals, respectively; 10 animals were used in total, n represents the number of cultures). Seizure incidence was slightly, but not significantly lower in cultures maintained in CBM compared to cultures maintained in NeurA during all time periods (Fig 8C and 8E; p = 0.141, 0.742, 0.769, 0.348 for 0–3, 4–7, 8–10, 11–14 DIV, respectively, z-test). We then examined seizure frequency, duration, and total time seizing per hour only in cultures with seizures. The only significant reduction in average seizure duration was found in CBM vs. NeurA during the 4–7 DIV time period (p = 0.039, n = 6 for NeurA, n = 5 for CBM). Thus, CBM cultures were somewhat less epileptic than NeurA cultures, which matches well with the differences in lactate between cultures maintained in CBM and NeurA media. However, electrical recordings also revealed that number of seizures per hour (p = 0.094 on 8–10 DIV, n = 6 for NeurA, n = 7 for CBM; p = 0.344 on 11–14 DIV, n = 8 for NeurA, n = 5 for CBM, n represents the number of cultures), and time seizing per hour (p = 0.201 on 8–10 DIV, n = 6 for NeurA, n = 7 for CBM; p = 0.713 on 11–14 DIV, n = 8 for NeurA, n = 5 for CBM) were slightly but not significantly lower in CBM compared to NeurA after 8 DIV. In cultures that had electrographic seizures, average seizure duration was actually slightly, but not significantly higher in CBM compared to NeurA at 11–14 DIV (p = 0.057, n = 8 for NeurA, n = 5 for CBM). Electrophysiology data is provided in S1 Spreadsheet.

### Epileptogenesis occurs independently of medium composition

**Fig 9:**
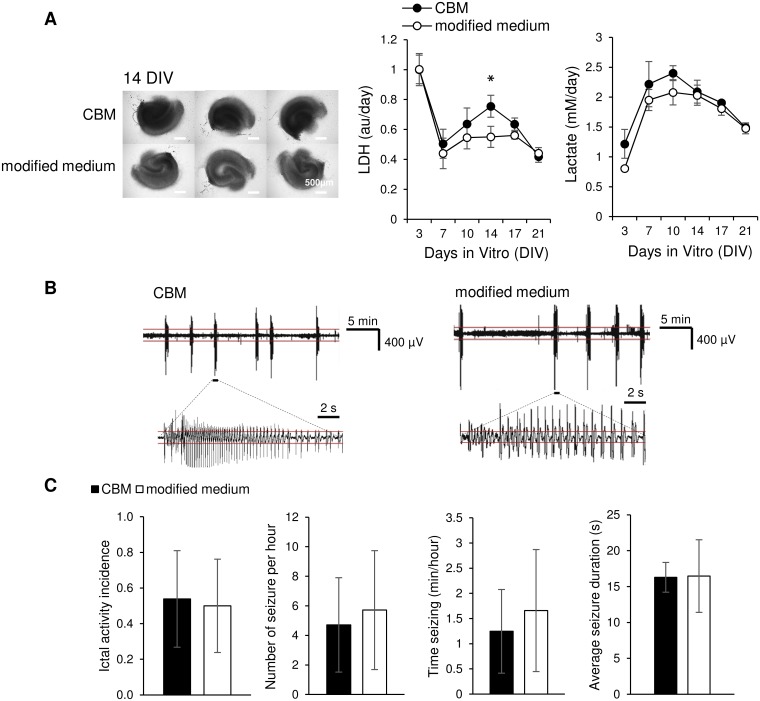
Epileptogenesis occurs independently of medium composition. (A) Left: brightfield images at 14 DIV. Modified medium refers to the medium with lower concentrations of glycine and serine (0.01 mM) and higher concentration of magnesium (2.0 mM) compared to CBM. Scale bars, 500 μm; Right: time course of LDH and lactate concentration; n = 3 cultures, each condition. Error bars indicate SD. *p < 0.05. (B) Representative recordings of electrographic seizure-like events in organotypic cultures maintained in CBM and modified medium on 11 DIV. Seizure detail is shown in lower traces. Red lines indicate the threshold for paroxysmal event detection. (C) Comparison of electrographic seizures in cultures maintained in CBM or modified medium during 10–14 DIV. n = 13 cultures for CBM; n = 14 cultures for modified medium; only cultures with seizures used for quantification of seizure frequency, duration, and time seizing. Error bars represent 95% confidence intervals for incidence of ictal activity and SD for other graphs.

Previous experiments revealed that reduction of glycine and L-serine concentrations to 0.01 mM and increase of [Mg^2+^] to 2 mM significantly decreased cell death and reduced ictal activity (Figs 6C and 7C). We integrated these two modifications into a modified medium, and compared it with CBM. Microscope images at 14 DIV revealed that cultures in modified media had significantly brighter neural layers than cultures in CBM (Fig 9A; inverted greyscale = 155.22 ± 8.37 and 134.15 ± 5.06 for CBM and modified medium group respectively, p = 0.038, n = 3 cultures, each condition). Cultures maintained in modified medium released significantly less LDH than slices in CBM at 14 DIV (p = 0.048, n = 3 cultures, each condition). No significant differences were observed in lactate release between two groups. We recorded electrographic activity in CBM and modified media cultures from 10 DIV to 14 DIV (Fig 9B and 9C; for CBM, n = 13 cultures from 3 animals; for modified medium, n = 14 cultures from 3 animals). No significant differences were observed in ictal activity incidence (p = 0.853, z-test). In cultures with seizures (n = 7 cultures for CBM and modified medium), no significant differences were observed in the number of seizure per hour, average seizure duration or time seizing (p = 0.642, p = 0.937, p = 0.506 respectively, *t* test). Electrophysiology data is provided in S2 Spreadsheet. It therefore appears that this modified medium was moderately neuroprotective (better morphology, lower LDH), but did not affect epileptogenesis.

## Discussion

### Interpretation of morphology, lactate, and LDH data

Slice dissection results in substantial cell death at the surface layers of hippocampal slices. Cell death in organotypic hippocampal cultures continues until 3 DIV, and then almost completely disappears by 7 DIV as dead cells are cleared from organotypic cultures maintained in serum-based or Neurobasal-A/B27 media [4]. Brightfield microscope images taken at 3 DIV show relatively dark but clearly distinguishable neural layers, while images taken at 7 DIV show much brighter neural layers. Measurement of LDH in the culture medium at 3 DIV (capturing LDH release between 0 and 3 DIV) reveals much higher LDH concentration (corresponding to higher cell death [51]) than at 7 DIV (LDH release between 3 and 7 DIV) [4]. From 7 to 14 DIV, the incidence of spontaneous electrographic seizures increases in organotypic hippocampal cultures maintained in serum-based or Neurobasal-A media [3, 4]. Spontaneous seizures are accompanied by activity-dependent neuron death, which results in darker, less distinguishable neural layers by 10–14 DIV. The glutamate receptor antagonist kynurenic acid and anticonvulsant phenytoin reduce occurrence of spontaneous seizures and accompanying cell death. Cultures maintained in the presence of kynurenic acid or phenytoin have clearer neural layers and release less LDH into spent culture media between 10–14 DIV than vehicle-treated cultures [4, 52]. Thus, in cultures maintained in serum-based or Neurobasal-A media, morphology and LDH release are closely linked, and follow a predictable pattern: darker cultures and high LDH release signifying high cell death between 0 and 3 DIV, brighter, clearer cultures, low LDH release, and low cell death, between 3 and 7 DIV, and darker cultures and increasing LDH release accompanying seizure-induced cell death after 7 DIV. Lactate release into culture medium is correlated to the amount of time spent cultures seizing per hour [5]. Thus, little lactate is released into culture medium until 7 DIV, while after 7 DIV, increasing incidence of seizures causes increasing lactate release.

We found that changes in medium composition can negatively affect culture survival, and reduce the correlation between morphology and LDH release. For example, exclusion of vitamins in culture medium did not affect LDH release, but caused significant changes in morphology (Fig 5F). This may occur because LDH release from dead cells is affected by the mode of cell death: loss of membrane integrity in necrotic cells results in release of LDH into culture supernatant, while most of LDH in apoptotic cells may be degraded without release into extracellular space [43]. Therefore, we used culture morphology as first determinant of culture survival in different media, while LDH release was used to gauge culture survival when no differences were detected morphologically. When a change in medium composition resulted in decreased culture survival, lactate release was also decreased (effects of 0.01x insulin in Fig 3C or 4 mM Ca^2+^ in Fig 7B). This decrease occurred due to fewer surviving cells present in the culture to release lactate, and not necessarily due to lower seizure load. Thus, lactate, LDH, and morphology data needed to be analyzed together to determine effects of a medium composition on culture survival and development of epilepsy.

### Effect of medium composition on epileptogenesis

We investigated the influence of BSA, selenium and insulin on culture survival. Albumin may play a role in epileptogenesis by triggering TGF-β pathway activation after blood-brain barrier is compromised [38, 53]. However, we did not observe any significant differences in morphology, LDH or lactate in cultures maintained in various concentrations of BSA. Selenium is an antioxidant trace element that is protective against oxidative damage and can prevent the development of epilepsy induced by peroxidative injury [54–56]. Our experiments confirmed that selenium is essential to culture survival, but increasing the concentration of selenium had no beneficial or harmful effects. Insulin is frequently used in serum-free media formulations to stimulate cell growth [57], and has been reported to prevent apoptosis of external granular layer neurons in rat cerebellar slice cultures [58]. Our experiments confirmed that insulin affects neuronal size and is essential for neuronal survival (Fig 4). We therefore supplemented all media used in subsequent experiments with insulin and selenium to promote neuronal survival, and with BSA concentration that was in the range of albumin concentration in normal CSF.

We attempted to change concentrations of essential and non-essential amino acids, vitamins, electrolytes, and glucose to bring culture medium composition closer to CSF composition. We found that changing concentrations of essential amino acids, or removing some essential amino acids, negatively affected culture survival. Similarly, we found that organotypic cultures could not survive without vitamins. On the other hand, we found that cultures were more tolerant of changes in concentrations of non-essential amino acids, electrolytes, and glucose. Alanine and proline could be removed from culture medium, while concentrations of glycine and serine could be varied from 10 to 400 μM without affecting culture viability. We also found that concentration of glucose in the range of 11–25 mM, [Ca^2+^] between 1–2 mM, [Mg^2+^] between 0.5–4 mM, and [K^+^] between 3.5–8.5 mM supported culture survival.

In healthy CSF, the concentration of K^+^ is 2.7–3.9 mM, the concentration of Mg^2+^ is 1–2 mM and the concentration of Ca^2+^ is 1.5–2.5 mM [26, 29, 31]. Changes in the concentrations of these ions can alter neuronal excitability and lead to spontaneous epileptiform activity [26, 31, 47, 59–62]. Glucose concentration in CSF is approximately 1.5–5 mM, although 10–11 mM concentrations are used in acute slice preparations to compensate for the altered glucose delivery pathway: rather than active transport from capillaries, glucose is provided via the diffusion gradient between the bathing medium and the extracellular fluid of actively metabolizing cells [31, 50]. CSF contains 25–300 μM of serine and 5–40 μM of glycine depending on age [32, 33, 63]. Glycine affects excitatory neurotransmission by modulating N-methyl-d-aspartate (NMDA) receptor activation and desensitization [64–66], activates glycine receptors, and alters induced epileptiform activity [67]. L-serine (contained in culture medium) serves as a precursor for the synthesis of neuromodulators glycine and D-serine [68], which is also a co-agonist for the majority of NMDA receptor subtypes [66, 69]. Finally, L-serine and glycine can act as trophic factors for survival and dendritogenesis of neurons [70].

We created two CSF-based media, CBM and modified CBM, to examine whether epileptogenesis in organotypic cultures is caused by medium composition. Comparison between CBM and Neurobasal-A revealed that both media supported culture survival equally well, with a trend toward lower excitability in CBM cultures relative to Neurobasal-A cultures. This may be due to lower [K^+^] and glucose in CBM. However, the incidence of electrographic seizures was the same in both media, leading us to conclude that effect on epileptogenesis was minor. In modified CBM, we increased [Mg^2+^] to 2 mM, and reduced glycine and serine concentrations to 10 μM in an effort to minimize excitability but keep medium components within physiological ranges (note that Mg^2+^ was more protective at 4 mM, but this concentration is higher than what is found in CSF). Reducing glycine concentrations from 400 μM to 10 μM, which is slightly below the point of saturation for NMDA receptors [71], and increasing [Mg^2+^] from 1.3 mM to 2 mM may reduce Ca^2+^ entry through the NMDA receptors during seizures, thus lowering excitotoxicity [72]. However, electrophysiological recordings revealed that there were no changes in seizure incidence or the total time seizing; in other words, epileptogenesis was not altered in cultures maintained in medium that was formulated for low excitability. On the other hand, culture survival was slightly improved.

## Conclusions

Changes to culture medium composition moderately reduced electrographic seizure load and decreased cell death in organotypic hippocampal cultures. However, epilepsy developed in all media compositions that supported neuronal survival. Thus, medium composition is unlikely to be the cause of epileptogenesis in the organotypic hippocampal culture model of epilepsy.

## Supporting information

S1 Spreadsheet NeurA vs CBM field recordings. (XLSX)

S2 Spreadsheet CBM vs modified medium field recordings. (XLSX)
